# Chirurgische Therapie des Adenokarzinoms des ösophagogastralen Übergangs Typ II: Vergleich zwischen transhiatal erweiterter Gastrektomie und thorakoabdomineller Ösophagektomie

**DOI:** 10.1007/s00104-022-01703-x

**Published:** 2022-08-20

**Authors:** Roman Stürzl, Michael Gerken, Christiane Bruns, Monika Klinkhammer-Schalke, Armin Pauer, Pompiliu Piso

**Affiliations:** 1 Tumorzentrum, Institut für Qualitätssicherung und Versorgungsforschung, Universität Regensburg, Franz-Josef-Strauß-Allee 11, 93053 Regensburg, Deutschland; 2 Bayrisches Krebsregister, Regionalzentrum Regensburg, Bayerisches Landesamt für Gesundheit und Lebensmittelsicherheit, Regensburg, Deutschland; 3 Klinik für Allgemein‑, Viszeral‑, Tumor- und Transplantationschirurgie, Universitätsklinikum Köln, Köln, Deutschland; 4 Klinik für Allgemein- und Viszeralchirurgie, Krankenhaus Barmherzige Brüder Regensburg, Regensburg, Deutschland

**Keywords:** AEG Typ II, Siewert-Klassifikation, Cardia, Transhiatal erweiterte Gastrektomie, Thorakoabdominelle Ösophagektomie, AEG type II, Siewert classification, Cardia, Transhiatal extended gastrectomy, Thoracoabdominal esophagectomy

## Abstract

**Hintergrund:**

Trotz steigender Inzidenz ist die optimale chirurgische Versorgung des Adenokarzinoms des ösophagogastralen Übergangs (AEG) Typ II weiterhin Gegenstand aktueller Forschung. Ziel dieser Untersuchung ist es, Überlebens- und Rezidivraten von Patienten zu vergleichen, die sich entweder einer transhiatal erweiterten Gastrektomie (TEG) oder einer thorakoabdominellen Ösophagektomie (TAE) unterzogen.

**Material und Methoden:**

Es handelt es sich um eine retrospektive populationsbasierte Kohortenstudie (*n* = 272) auf der Basis von Krebsregisterdaten. Berücksichtigt wurden alle Patienten, die zwischen 2002 und 2020 an einem AEG Typ II erkrankten. Während 63 Patienten mittels TAE operiert wurden, gehören der Gruppe der TEG 209 Patienten an. Um das Gesamtüberleben, Rezidivraten und rezidivfreies Überleben zu untersuchen, wurden die Kaplan-Meier-Methode sowie uni- und multivariable Cox-Regressionen angewendet.

**Ergebnisse:**

Zwischen beiden Operationsgruppen gab es bezüglich des Gesamtüberlebens keinen signifikanten Unterschied (*p* = 0,333). Allerdings ließ die Richtung der HR im Zeitraum von 2016 bis 2020 eine mögliche Tendenz in Richtung der TAE erkennen (*p* = 0,058). Im Gegensatz dazu ergab der Kaplan-Meier-Schätzer ein signifikant höheres Risiko, nach einer TAE an einem Rezidiv zu erkranken (*p* = 0,049). Dies konnte nicht im Zeitraum 2016 bis 2020, der mehr als die Hälfte der TAE-Patienten umfasst, beobachtet werden (*p* = 0,993). Hinsichtlich des rezidivfreien Überlebens wurden keine signifikanten Unterschiede detektiert (*p* = 0,772).

**Diskussion:**

Die Ergebnisse dieser verhältnismäßig kleinen Kohorte decken sich mit den meisten Studien, die hinsichtlich des Gesamtüberlebens entweder keine Unterschiede oder eine Tendenz in Richtung der TAE fanden. Weitere Arbeiten kamen ebenfalls zu dem Ergebnis, dass es keine signifikanten Unterschiede zwischen den beiden Operationsgruppen bezüglich des rezidivfreien Überlebens gibt. Zusammenfassend lässt sich festhalten, dass es keine signifikanten Unterschiede zwischen TEG und TAE in der Therapie des AEG Typ II gibt.

## Abstract (en)

**Background:**

The incidence of adenocarcinoma of the esophagogastric junction (AEG) has significantly increased. However, the best surgical treatment for AEG type II is still the subject of current research. The goal of this retrospective cohort study is to compare survival and recurrence rates in patients, who underwent either thoracoabdominal esophagectomy (TAE) or transhiatal extended gastrectomy (TEG).

**Material and methods:**

The study is based on a cohort of 272 patients diagnosed with AEG type II between 2002 and 2020, recorded by a population-based clinical cancer registry. Of the included patients 63 underwent TAE and 209 TEG. In order to compare overall survival, recurrence rates, and recurrence free survival, we applied the Kaplan-Meier method, univariable and multivariable Cox regression.

**Results:**

Our analysis showed no statistically significant difference concerning overall survival (*p* = 0.333). However a tendency towards higher survival rates after TAE for the period from 2016–2020 (*p* = 0,058) is possible. In contrast a significant difference concerning higher cumulative recurrence rates after TAE was found after Kaplan-Meier analysis (*p* = 0.049). This trend was not observed for the time after 2016 (*p* = 0.993), in which over 50% of TAE were performed. No differences were found regarding recurrence-free survival (*p* = 0.772).

**Conclusion:**

Our findings in a rather small cohort are concordant with most studies showing no differences or a trend towards better survival after TAE. Other studies found no significant difference regarding recurrence-free survival as well. In conclusion, no significant differences were found between TEG and TAE in surgical treatment of AEG type II.

In den vergangenen Jahren hat die Inzidenz von Adenokarzinomen des ösophagogastralen Übergangs signifikant zugekommen. Die chirurgische Resektion stellt bei dieser Tumorentität im nichtmetastasierten Stadium die einzige kurative Therapiemöglichkeit dar und nimmt daher in der Behandlung der AEG einen besonderen Stellenwert ein. Das sogenannte Kardiakarzinom, welches dem Typ II nach Siewert entspricht, kann aus operationstechnischer Sicht mittels transhiatal erweiterter Gastrektomie oder thorakoabdomineller Ösophagektomie reseziert werden. In diesem Beitrag werden daher die beiden Operationsmethoden bezüglich des Outcomes der Patienten untersucht und miteinander verglichen.

## Hintergrund und Fragestellung

In Deutschland konnte in den vergangenen Jahren eine Abnahme der Erkrankungs- und der Sterberaten von Magenkarzinomen verzeichnet werden [1]. Dieser Trend wurde ebenfalls für Adenokarzinome des Magens oder Ösophagus beobachtet. Gänzlich anders hingegen stellt sich die Situation bei Adenokarzinomen des ösophagogastralen Übergangs (AEG) dar. Die Inzidenz dieser eigenen Tumorentität hat über die Jahre signifikant zugenommen, weshalb deren chirurgische Therapie im klinischen Alltag von immer größerer Bedeutung ist [2].

**Figure 1:**
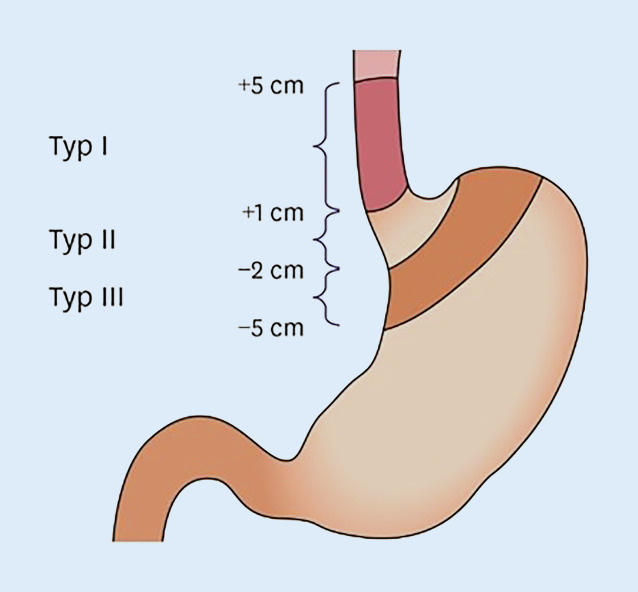

Zur genaueren Differenzierung von AEG gilt die Klassifikation nach Siewert (Abb. 1) heutzutage als Standardklassifikation in westlichen Ländern. Hierbei dient die Z‑Linie als wichtige anatomische Landmarke für die Einteilung in die Typen I bis III. Sie markiert den histologischen Übergang zwischen dem Epithel von Ösophagus und Magen. Befindet sich das Zentrum des Tumors 1–5 cm oberhalb der Z‑Linie spricht man vom Typ I. Als Typ II werden AEG klassifiziert, deren Haupttumormasse in einem Bereich 1 cm oberhalb bis 2 cm unterhalb der Z‑Linie liegen. Aufgrund seiner Lage wird ein AEG Typ II auch als Kardiakarzinom oder „true junctional cancer“ bezeichnet. Alle Adenokarzinome in einem Bereich 2–5 cm unterhalb der Z‑Linie werden als Typ III zusammengefasst [3].

Die Siewert-Klassifikation ist darüber hinaus maßgebend für die Wahl der Operationsmethode und somit für die Therapie. Sowohl für AEG Typ I als auch für den Typ III gibt es aufgrund von Studien klar präferierte Operationsmethoden. Bei einem AEG Typ I wird eine subtotale transthorakale Ösophagektomie mit Resektion des proximalen Magens durchgeführt. Beim AEG Typ III hingegen stellt die transhiatal erweiterte Gastrektomie mit distaler Ösophagusresektion das chirurgische Standardverfahren dar. Ein AEG Typ II kann aus chirurgisch-technischer Sicht nach beiden Methoden reseziert werden. Trotz verschiedener Studien konnte die Frage, ob eine der Operationsmethoden überlegen ist, bis heute nicht abschließend geklärt werden. Daher werden erstmals in Deutschland im Rahmen einer populationsbasierten retrospektiven Kohortenstudie die beiden Operationsmethoden, die in der Therapie des AEG Typ II Anwendung finden, untersucht und bezüglich folgender Outcomeparameter miteinander verglichen:

- Verteilung der Operationsmethoden,
- Operationskomplikationen,
- postoperative Mortalität,
- Rezidivraten,
- Gesamtüberleben,
- rezidivfreies Überleben.

## Studiendesign und Untersuchungsmethoden

### Studiendesign

Bei der vorliegenden Arbeit handelt es sich um eine retrospektive populationsbasierte Kohortenstudie basierend auf Krebsregisterdaten. Die Grundgesamtheit umfasst alle Patienten mit der Erstdiagnose einer bösartigen Neubildung der Kardia des Magens (C16.0), deren Wohnsitz sich in der Oberpfalz oder Niederbayern befindet. Dabei wurden sämtliche Diagnosen, die im Zeitraum vom 01.01.2002 bis zum 31.12.2020 gestellt wurden, berücksichtigt. Die Datenbasis der Studie bildet sich dabei aus den Angaben in der Tumordokumentationsdatenbank des Regionalzentrums Regensburg des Bayerischen Krebsregisters. Dabei wurden Arztbriefe, Operationsprotokolle, histologische Befunde etc. verwendet sowie bei fehlenden Angaben einzelne Dokumente direkt bei den Krankenhäusern nachgefordert. Die Endpunkte der Untersuchung waren Gesamtüberleben, kumulative Rezidivraten und rezidivfreies Überleben. Diese wurden neben der Analyse im Gesamtkollektiv innerhalb der Subgruppe der Patienten mit Diagnosedatum 2016 bis 2020 analysiert.

### Statistische Methoden und Analysen

Zur Darstellung der Verteilung von Einflussvariablen wurden Kontingenztabellen verwendet. Diese enthalten neben den absoluten Werten stets auch prozentuale Angaben (%). Um Gruppen hinsichtlich kategorialer Variablen zu vergleichen und auf statistische Unabhängigkeit zu überprüfen, wurde der χ^2^-Test angewandt.

Zur Untersuchung der perioperativen Mortalität wurde der absolute und relative Anteil der Patienten angegeben, die in einem Zeitraum von 30 Tagen nach der Operation verstarben.

Um die genannten Endpunkte zu ermitteln, wurde die Differenz zwischen dem Datum der primären Operation und dem ersten Ereignis, je nach Analyse Rezidiv und/oder Tod, berechnet. Die verwendeten Angaben zum Life- bzw. Rezidivstatus basieren auf Todesbescheinigungen der Gesundheitsämter, den klinischen Unterlagen und auf personenbezogenen Abfragen bei den zuständigen Einwohnermeldeämtern. Dabei wurde ein Rezidiv als neu aufgetretener Tumor definiert, der histologisch auf das ursprüngliche AEG Typ II zurückzuführen ist, unabhängig davon, ob ein Lokal‑, Lymphknoten- oder Fernmetastasenrezidiv vorlag. Konnte dabei für einen Patienten kein Ereignis festgestellt werden, so wurde anstelle des Datums des Ereignisses das letzte bekannte Datum, zu dem der Patient noch lebte, verwendet.

Die Endpunkte wurden mithilfe der Kaplan-Meier-Methode geschätzt. Zudem wurde das relative Sterbe- bzw. Rezidivrisiko (Hazard Ratio [HR]) mit Cox-Regressionsanalysen geschätzt. Das dazugehörige 95 %-Konfidenzintervall (95 %-KI) sowie der *p*-Wert wurden mitangegeben. Bei multivariablen Cox-Regressionen erfolgte eine Risikoadjustierung für Variablen mit potenziellem Einfluss auf Überleben bzw. Rezidivhäufigkeit. Dazu gehörten die Variablen Geschlecht, Diagnosealter, Diagnosejahr, histologischer Typ des AEG, Primärtumor (pT), regionale Lymphknoten (pN), Grading, Lymphgefäßinvasion, Veneninvasion und primäre Chemotherapie. Um ausreichend hohe Fallzahlen zu gewährleisten, wurden teilweise mehrere Gruppen einer Variablen zusammengefasst, wie in Tab. 1 ersichtlich.

**Table 1:** 

–		Multivariable Cox-Regression			
Kategorie	Gruppe	HR	p	Unteres 95 %-KI	Oberes 95 %-KI
Primäre Operation	TEG	1,000	–	–	–
	TAE	0,790	0,333	0,491	1,272
Chemotherapie	Nein	1,000	–	–	–
	Neo-/adjuvant	0,875	0,515	0,584	1,309
Geschlecht	Männlich	1,000	–	–	–
	Weiblich	0,982	0,932	0,651	1,482
Diagnosealter	< 60,0 Jahre	1,000	–	–	–
	≥ 60,0 Jahre	1,392	0,106	0,932	2,078
Diagnosejahr	2002–2005	1,000	–	–	–
	2006–2010	1,424	0,161	0,869	2,335
	2011–2015	1,499	0,197	0,810	2,773
	2016–2020	1,641	0,159	0,823	3,270
Histologie	AEG o.n.A/diffus/intestinal	1,000	–	–	–
	Andere AEG	0,582	0,063	0,329	1,030
pT	T1	1,000	–	–	–
	T2	1,443	0,172	0,853	2,441
	T3/4	2,098	0,005	1,245	3,534
pN	N0	1,000	–	–	–
	N+	1,900	0,002	1,275	2,832
Grading	G1/2	1,000	–	–	–
	G3/4	1,361	0,127	0,916	2,022
	GX/kA	1,521	0,451	0,511	4,524
Lymphgefäßinvasion	L0	1,000	–	–	–
	L1	1,552	0,075	0,957	2,518
	LX/kA	1,506	0,398	0,583	3,891
Veneninvasion	V0	1,000	–	–	–
	V1	0,929	0,808	0,515	1,678
	VX/kA	0,871	0,733	0,394	1,925

*o.n.A.* ohne nähere Angaben, *kA* keine Angabe, *HR* Hazard Ratio, *KI* Konfidenzintervall

## Ergebnisse

### Ausschlusskriterien

**Figure 2:**
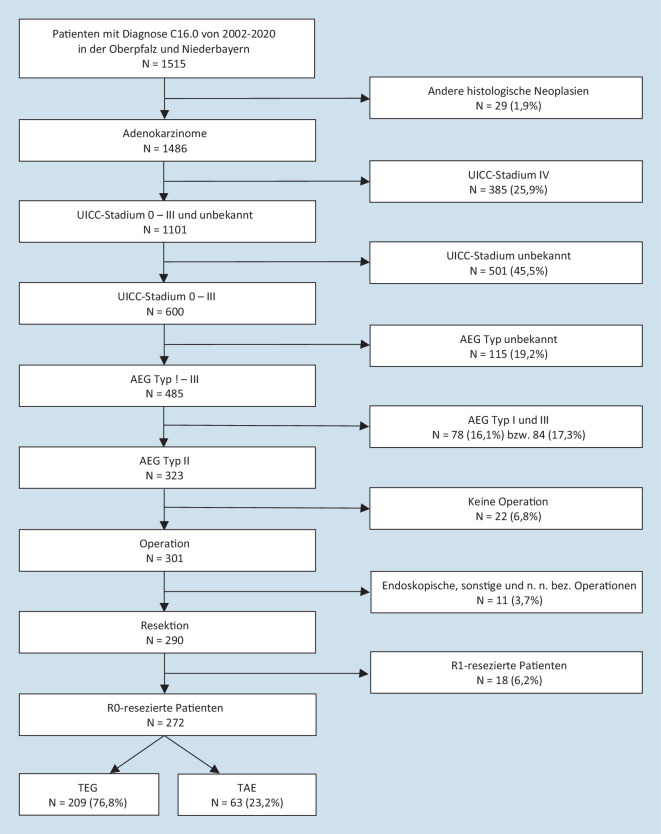

Das Auswertekollektiv besteht aus allen Patienten, bei denen ein AEG Typ II nach Siewert mittels Gastroskopie diagnostiziert wurde (Haupttumormasse 1 cm oberhalb bis 2 cm unterhalb der Z‑Linie). Andere histologische Neoplasien wurden ausgeschlossen. Darüber hinaus wurden Patienten mit UICC-Stadium IV oder unbekannt exkludiert. Patienten mit einem AEG Typ I, III oder unbekanntem AEG-Typ wurden nicht berücksichtigt. Zudem musste eine R0-Resektion des Tumors in Form einer transhiatal erweiterten Gastrektomie oder thorakoabdomineller Ösophagektomie erfolgen. Alle Patienten, die eine endoskopische oder nicht näher bezeichnete Operation erhielten, wurden ebenfalls ausgeschlossen (Abb. 2).

### Patientencharakteristika

Die Verteilung der wichtigsten Patienten- und Tumorcharakteristika nach Operationsmethode zeigt Tab. 2.

**Table 2:** 

		TEG		TAE		Gesamt	
		Anzahl	(%)	Anzahl	(%)	Anzahl	(%)
Geschlecht	Männlich	153	73,2	53	84,1	206	75,7
	Weiblich	56	26,8	10	15,9	66	24,3
Diagnosealter	< 60,0 Jahre	60	28,7	25	39,7	85	31,3
	≥ 60,0 Jahre	149	71,3	38	60,3	187	68,8
Diagnosejahr	2002–2005	41	19,6	7	11,1	48	17,6
	2006–2010	53	25,4	6	9,5	59	21,7
	2011–2015	66	31,6	16	25,4	82	30,1
	2016–2020	49	23,4	34	54,0	83	30,5
Histologie	AEG o.n.A./diffus/intestinal	179	85,6	54	85,7	233	85,7
	Andere AEG	30	14,4	9	14,3	39	14,3
pT	T1	57	27,3	19	30,1	76	28,0
	T2	60	28,7	19	30,2	79	29,0
	T3	84	40,2	24	38,1	108	39,7
	T4	8	3,8	1	1,6	9	3,3
pN	N0	107	51,2	39	61,9	146	53,7
	N1	63	30,1	14	22,2	77	28,3
	N2	28	13,4	5	7,9	33	12,1
	N3	11	5,3	5	7,9	16	5,9
Stadium	I	45	21,5	16	25,4	61	22,4
	II	61	29,2	13	20,6	74	27,2
	III	103	49,3	34	54,0	137	50,4
Grading	G1	2	1,0	3	4,8	5	1,8
	G2	76	36,4	28	44,4	104	38,2
	G3	124	59,3	27	42,9	151	55,5
	GX/kA	7	3,3	5	7,9	12	4,4
Lymphgefäßinvasion	L0	87	41,6	34	54,0	121	44,5
	L1	80	38,3	20	31,7	100	36,8
	LX/kA	42	20,1	9	14,3	51	18,8
Veneninvasion	V0	131	62,7	51	81,0	182	66,9
	V1	23	11,0	3	4,8	26	9,6
	VX/kA	55	26,3	9	14,3	64	23,5
Chemotherapie	Nein	89	42,6	27	42,9	116	42,6
	Ja – neoadjuvant	113	54,1	36	57,1	149	54,8
	Ja – adjuvant	7	3,3	0	0,0	7	2,6
Gesamt		209	100,0	63	100,0	272	100,0

*o.n.A.* ohne nähere Angaben, *kA* keine Angabe

Etwa drei Viertel der Patienten gehören dem männlichen Geschlecht an (75,7 %; *n* = 206). Eine ähnliche Verteilung findet sich in beiden Operationsgruppen wieder (*p* = 0,076). Innerhalb der Gruppe der TEG befanden sich 73,2 % (*n* = 153) männliche Patienten, während es bei der TAE sogar 84,1 % (*n* = 53) waren.

Die Diagnose eines AEG Typ II wurde bei einem Großteil der Patienten im Alter > 60 Jahre gestellt (68,8 %; *n* = 187). Diese Tendenz in Richtung eines fortgeschrittenen Diagnosealters findet sich sowohl in der Gruppe der TEG als auch der TAE wieder (71,3 %; *n* = 149 bzw. 60,3 %; *n* = 38). Im gesamten Kollektiv betrug das mittlere Alter 65,1 Jahre, während das mediane Alter bei 66,0 Jahren lag. In der Gruppe der TEG lag der Mittelwert bzw. Median des Diagnosealters bei 66,1 bzw. 67,5 Jahren. Bei der TAE hingegen betrug das mittlere bzw. mediane Alter lediglich 61,6 bzw. 62,6 Jahre.

### Tumorcharakteristika

Bei der histologischen Differenzierung des AEG stellten Adenokarzinome o.n.A. (TEG: 43,1 %; *n* = 90 bzw. TAE: 69,8 %; *n* = 44) und Adenokarzinome vom intestinalen Typ (TEG: 34,0 %; *n* = 71 bzw. TAE: 12,7 %; *n* = 8) die beiden größten Gruppen dar.

Sowohl Innerhalb der TEG (40,2 %; *n* = 84) als auch der TAE (38,1 %; *n* = 24) wurden die meisten Karzinome mit einer Tumorgröße entsprechend T3 diagnostiziert. Während bei der TEG rund die Hälfte der Patienten einen positiven Lymphknotenbefall zeigten (48,8 %; *n* = 102), lag die Rate nach TAE ca. 10 % niedriger (38,1 %; *n* = 24). Auf das gesamte Kollektiv bezogen wurden im Durchschnitt 26 Lymphknoten zur Untersuchung entnommen, wobei im Mittel zwei befallen waren. Dementsprechend wird bezüglich des Stadiums ersichtlich, dass die Mehrzahl der Diagnosen (50,4 %; *n* = 137) erst im UICC-Stadium III gestellt wurden. Sowohl bei der TEG als auch der TAE zeigt sich ein ähnliches Bild (*p* = 0,400).

In beiden Gruppen lag der Anteil der Patienten mit L0-Status (TEG: 41,6 %; *n* = 87 bzw. TAE: 54,0 %; *n* = 34) höher als derer mit positiver Lymphgefäßinvasion (TEG: 38,3 %; *n* = 80 bzw. TAE: 31,7 %; *n* = 20). Der Großteil des Kollektivs zeigte zudem keine Veneninvasion durch das AEG (TEG: 62,7 %; *n* = 131 bzw. TAE: 81,0 %; *n* = 51). Allerdings konnte bei einer nicht unerheblichen Anzahl an Patienten der L‑Status (TEG: 20,1 %; *n* = 42 bzw. TAE: 14,3 %; *n* = 9) und der V‑Status (TEG: 26,3 %; *n* = 55 bzw. TAE: 14,3 %; *n* = 9) nicht erhoben werden.

### Verteilung der Chemotherapie

Im Rahmen der perioperativen Therapie erhielten bei der TEG 54,1 % (*n* = 113) und bei der TAE 57,1 % (*n* = 36) eine neoadjuvante Chemotherapie. Lediglich innerhalb der Gruppe der TEG (3,3 %; *n* = 7) erhielt ein geringer Anteil eine primär adjuvante Chemo. Dementsprechend erfolgte in beiden Gruppen bei knapp über 40 % der Patienten keine primäre Chemotherapie (TEG: 42,6 %; *n* = 89 bzw. TAE: 42,9 %; *n* = 27).

### Verteilung der Operationsmethoden

Aus dem Kollektiv (*n* = 272) erhielten mit 76,8 % (*n* = 209) mehr als drei Viertel der Patienten eine transhiatal erweiterte Gastrektomie. Bei 23,2 % (*n* = 63) der Patienten wurde der Tumor mittels thorakoabdomineller Ösophagektomie reseziert. Innerhalb der Gruppe der TEG stellte die „(totale) Gastrektomie“ (OPS: 5‑437) mit 75,1 % (*n* = 157) die mit Abstand häufigste Operation dar. Bei dieser Operation erfolgt intraoperativ eine Mitresektion einer Ösophagusmanschette von weniger als 4 cm. Unter den TAE wurde die „(totale) Ösophagektomie mit Wiederherstellung der Kontinuität“ (OPS: 5‑426) mit 79,4 % (*n* = 50) am öftesten angewendet. Dabei wird in der Regel ein Rekonstruktionsverfahren mittels Magenschlauchbildung und intrathorakaler Anastomose angewandt.

**Figure 3:**
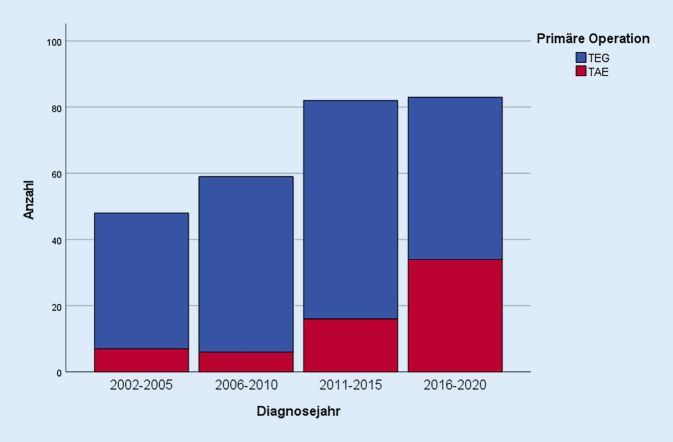

Allerdings ist diese Verteilung nicht über den gesamten Zeitraum hinweg zu beobachten. Daher wurden die Patienten in vier Zeiträume unterteilt und anhand derer die Verteilung der Operationsmethoden im zeitlichen Verlauf untersucht (Abb. 3).

In den Jahren 2002 bis 2010 spielte die TAE noch eine untergeordnete Rolle. Im Zeitraum 2002 bis 2005 (*n* = 48) lag ihr Anteil gerade einmal bei 14,6 % (*n* = 7). In den Jahren 2006 bis 2010 (*n* = 59) entfielen sogar nur 10,2 % (*n* = 6) der Operationen auf die TAE. Im darauffolgenden Zeitraum von 2011 bis 2015 (*n* = 82) kam es erstmal zu einer leichten Zunahme (19,5 %; *n* = 16). Dieser Trend setzte sich in den Jahren 2016 bis 2020 (*n* = 83) fort. In diesen Jahren erhielten 41,0 % (*n* = 34) der operierten Patienten eine TAE. Die Verteilung der TEG zeigt dazu passend ein gegensätzliches Bild. Die transhiatal erweiterte Gastrektomie galt Anfang der 2000er als standmäßige Operationsmethode des AEG Typ II. In den ersten beiden Zeiträumen wurde sie deutlich häufiger angewendet als die TAE (85,4 %; *n* = 41 bzw. 89,8 %; *n* = 53). In den Jahren 2011 bis 2015 fand ein Umdenken stattfand, weshalb es in diesem Zeitraum erstmalig zu einer Abnahme des Anteils der TEG (80,5 %; *n* = 49) kam. Kongruent zu der beschriebenen Zunahme der TAE ab dem Jahr 2016 kam es im letzten Zeitraum der Untersuchung erneut zu einer Abnahme der TEG. In den Jahren 2016 bis 2020 erfolgten nur noch 59,0 % (*n* = 49) der Operationen in Form einer TEG. Es lässt sich festhalten, dass die Anzahl der resezierten AEG Typ II zugenommen hat und in den letzten Zeiträumen in etwa konstant geblieben ist. Parallel dazu erfolgte im zeitlichen Verlauf eine absolute und relative Zunahme der thorakoabdominellen Ösophagektomien.

### Operationskomplikationen

Insgesamt traten im Kollektiv bei 21,3 % (*n* = 58) mindestens eine dem Eingriff geschuldete Komplikation auf. Dieser Anteil ist in beiden Gruppen in etwa gleich groß und liegt bei den TEG bei 22,0 % (*n* = 46), während nach TAE bei 19,0 % (*n* = 12) der Patienten Komplikationen auftraten.

Unabhängig von der Wahl der Operationsmethode stellten Anastomoseninsuffizienzen in beiden Gruppen die häufigsten Komplikationen dar. Eine solche Leckage im Bereich des Gastrointestinaltrakts ist die klinisch relevanteste Form der Operationskomplikationen, da sie am häufigsten auftritt und als schwerwiegendste septische Komplikation viszeralchirurgischer Eingriffe gilt [4]. In der Gruppe der TEG trat bei 15,3 % (*n* = 32) eine Anastomoseninsuffizienz auf. Entsprechend dem oben beschriebenen Trend war diese Komplikation bei TAE mit 14,3 % (*n* = 9) nur geringfügig seltener (*p* = 0,510).

### Postoperative Mortalität

Zur Untersuchung der postoperativen Mortalität wurde ein Beobachtungszeitraum von 30 Tagen gewählt.

Während dieser Zeit verstarben insgesamt 2,6 % (*n* = 7) der Patienten. In der Gruppe der TEG liegt das postoperative Mortalitätsrisiko bei 2,9 % (*n* = 6), während es nach einer TAE lediglich 1,6 % (*n* = 1) beträgt. Zwar ist die Mortalität nach TAE leicht niedriger, allerdings liegen bei dieser Untersuchung in der Gruppe mit *n* = 1 auch eine sehr kleine Fallzahl vor. Zusammenfassend lässt sich also kein signifikanter Unterschied bezüglich der postoperativen Mortalität innerhalb der ersten 30 Tage zwischen den beiden Operationsgruppen erkennen (*p* = 0,573).

### Gesamtüberleben

Im gesamten Kollektiv lag die 5‑Jahres-Überlebensrate bei 42,8 %. Sowohl die univariable Cox-Regression als auch die Kaplan-Meier-Analyse ergaben keinen nennenswerten Unterschied zwischen den beiden Gruppen (*p* = 0,590). So lag die 5‑Jahres-Überlebensrate in der Gruppe der TEG bei 42,0 %, während sie nach TAE mit 46,4 % nur leicht höher lag. Die multivariable Cox-Regression zeigte keinen signifikanten Unterschied bezüglich des Überlebens (HR = 0,790; *p* = 0,333; 95 %-KI: 0,491–1,272). Als unabhängige Risikofaktoren für das Gesamtüberleben stellten sich die Variablen pT3/4 (HR = 2,098; *p* = 0,005; 95 %-KI: 1,245–3,534) sowie pN^+^ (HR = 1,900; *p* = 0,002; 95 %-KI: 1,275–2,832) heraus. Eine neo-/adjuvante Chemotherapie erwies sich hingegen als nicht signifikante Einflussgröße (Tab. 1) auf das Überleben (HR = 0,875; *p* = 0,515; 95 %-KI: 0,584–1,309).

### Rezidivraten

**Figure 4:**
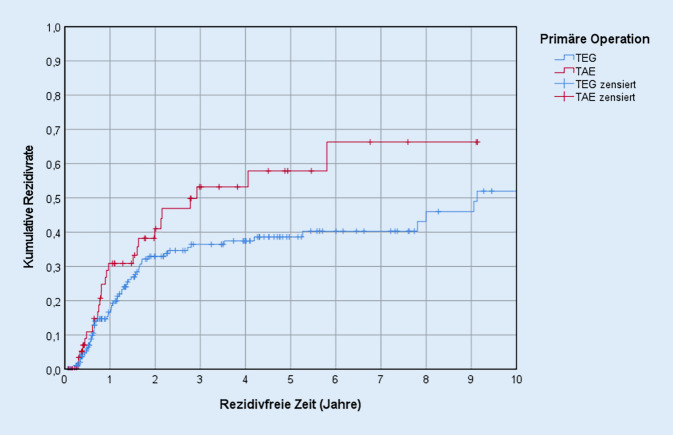

Insgesamt lag die kumulative 5‑Jahres-Rezidivrate bei 42,8 %. Sowohl der Kaplan-Meier-Schätzer (*p* = 0,049; Abb. 4) als auch die univariable Cox-Regression (HR = 1,579; *p* = 0,049; 95 %-KI: 0,997–2,498) zeigten, dass hierbei ein statistisch signifikanter Unterschied zwischen den beiden Operationsgruppen vorliegt, wobei die Patienten der TAE-Gruppe ein signifikant höheres Risiko haben, im Laufe ihres Lebens ein Rezidiv zu entwickeln.

Die multivariable Cox-Regression konnte diese Tendenz hinsichtlich niedrigerer Rezidivraten nach TEG zwar bestätigen, allerdings lag danach kein signifikanter Unterschied mehr vor (*p* = 0,201).

### Rezidivfreies Überleben

Insgesamt lag die Wahrscheinlichkeit, dass eines der Ereignisse Tod bzw. Rezidiv innerhalb der ersten 5 Jahre nach der Operation eintritt, bei 36,9 %. Der Vergleich der beiden Gruppen ergab, dass es zwischen diesen keinen statistisch signifikanten Unterschied gibt (*p* = 0,910).

Sowohl die univariable (HR = 1,023; *p* = 0,910; 95 %-KI: 0,692–1,513) als auch die multivariable Cox-Regression (HR = 0,939; *p* = 0,772; 95 %-KI: 0,613–1,438) bestätigten, dass die TAE der TEG bezüglich des rezidivfreien Überlebens nicht unterlegen ist und diesbezüglich keine gravierenden Unterschiede zwischen beiden Methoden zu erkennen sind.

#### Subgruppenanalyse des Zeitraums 2016 bis 2020

Im Zeitraum von 2016 bis 2020 wurde mehr als die Hälfte der gesamten Ösophagektomien (52,2 %; *n* = 35) durchgeführt. Dabei sind perioperative Therapie sowie die im Detail angewendeten Operationstechniken am ehesten mit den aktuellen vergleichbar. Aufgrund der hohen Fallzahlen und, um einen annähernd gleichen Beobachtungszeitraum zu ermöglichen, wurde das Kollektiv dieses Zeitraums gesondert untersucht.

**Figure 5:**
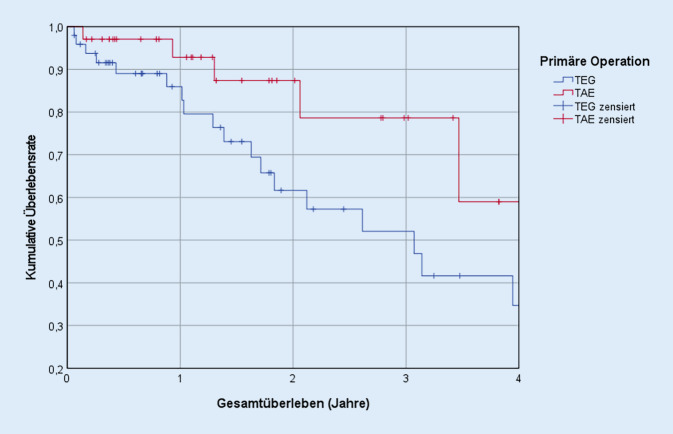

Auch in diesem Zeitraum lag zwischen beiden Gruppen kein statistisch signifikanter Unterschied (*p* = 0,058; HR = 0,299; 95 %-KI: 0,086–1,043) vor (Abb. 5). Allerdings lässt sich dabei eine mögliche Tendenz bezüglich eines besseren Gesamtüberlebens nach TAE erkennen.

Während das Rezidivrisiko auf das gesamte Kollektiv gesehen bei den Ösophagektomien signifikant höher lag, konnte dieser Nachteil innerhalb der Subgruppe nicht bestätigt werden. Für Patienten des Zeitraums 2016 bis 2020 gab es hinsichtlich des Risikos eines Rezidivs keine Unterschiede (*p* = 0,993).

Bezüglich des rezidivfreien Überlebens in diesem gesonderten Kollektiv war keine der beiden Operationsmethoden der anderen signifikant überlegen (*p* = 0,384; HR = 0,682; 95 %-KI: 0,289–1,614).

## Diskussion

Ziel dieser Arbeit war es, die transhiatal erweiterte Gastrektomie mit der thorakoabdominellen Ösophagektomie bei AEG-Typ-II-Patienten hinsichtlich des Gesamt- und rezidivfreien Überlebens sowie der kumulativen Rezidivraten zu vergleichen.

Traditionell wurde das AEG Typ II in der Vergangenheit in Deutschland mittels einer TEG operiert [5]. Internationalen Trends folgend fand auch hierzulande in den letzten zwei Dekaden ein stetiger Wandel statt. Gerade Thoraxchirurgen, die in den USA mehrheitlich Ösophaguskarzinome operieren, sahen nicht zuletzt aufgrund der eigenen Expertise einen Vorteil für die transthorakale Resektion [6]. Aber auch die aktuelle Leitlinie betrachtet die TEG und die TAE als gleichwertige Verfahren und empfiehlt beide für die Therapie des AEG Typ II [7].

Die Analyse der Patientencharakteristika ergab, dass ca. 75 % der Patienten des Kollektivs dem männlichen Geschlecht angehören. Dies deckt sich mit der Tatsache, dass Männer allgemein ein deutlich höheres Risiko tragen, im Laufe ihres Lebens an einem AEG zu erkranken. Nach Angaben in der Literatur liegt das Verhältnis zwischen Frauen und Männer mit 1:4,7 sogar etwas höher [2].

Zudem wurde gezeigt, dass die Mehrheit der Patienten ihre Diagnose erst im UICC-Stadium III bzw. bei einer Tumorgröße entsprechend T3 erhielt. Das liegt dran, dass die Dysphagie (Schluckstörung) als klassisches Leitsymptom der AEG oft erst in fortgeschrittenen Tumorstadien auftritt.

Sowohl bei der postoperativen Mortalität als auch bei den Operationskomplikationen, insbesondere Anastomoseninsuffizienzen, konnten keine signifikanten Unterschiede zwischen den beiden Gruppen festgestellt werden. Eine Metaanalyse, die diese beiden Punkte ebenfalls untersucht hat, kommt zu einem ähnlichen Ergebnis: Bezüglich der postoperativen Mortalität (30 Tage) konnten keine Unterschiede detektiert werden (*p* = 0,11). Dies gilt ebenso für das Risiko einer Anastomoseninsuffizienz (*p* = 0,63; [8]).

Erfreulicherweise war die Mortalität niedrig, gerade weil im bundesweiten Vergleich eine Letalität von 8,6 % nach Ösophagektomie und sogar 11,2 % nach Gastrektomie verzeichnet werden konnte [9].

Im vollständigen Kollektiv war das Gesamtüberleben betreffend keine Operationsmethode der anderen gegenüber im Nachteil (*p* = 0,330). Innerhalb der Subgruppe „Diagnosejahr 2016 bis 2020“ erlaubt die Richtung der HR die Vermutung, dass eine mögliche Tendenz des Gesamtüberlebens in Richtung der TAE vorliegen könnte (*p* = 0,058). Verschiedene Studien kommen ebenfalls zu dem Schluss, dass keine der beiden Operationen mit einem schlechteren Gesamtüberleben assoziiert ist [10–12]. Jedoch detektierten Parry et al. eine Tendenz bezüglich der 5‑Jahres-Überlebensrate zugunsten der Ösophagektomie (*p* = 0,05; [13]). Noch ausgeprägter fiel dieser Effekt bei Blank et al. aus. Diese fanden bei den 5‑Jahres-Überlebensraten einen signifikanten Unterschied zwischen den Gruppen (G = 38,8 %; Ö = 57,5 %; *p* = 0,02; [14]).

Die Analyse des rezidivfreien Überlebens ergab, dass keine der Operationsmethoden der anderen diesbezüglich unterlegen ist (*p* = 0,772). Parry et al. fanden hinsichtlich der rezidivfreien 5‑Jahres-Überlebensraten ebenfalls keinen Unterschied zwischen den Operationsmethoden (*p* = 0,25; [13]). Zu demselben Ergebnis (*p* = 0,40) kamen Schuhmacher et al. bei der Untersuchung der rezidivfreien 3‑Jahres-Überlebensrate [15]. Im Gegensatz dazu stellten Blank et al. einen signifikanten Vorteil (*p* = 0,002) zugunsten der TAE fest. Die rezidivfreie 5‑Jahres-Überlebensrate lag bei 79,1 %, während sie nach TEG 44,8 % betrug [14].

### Limitation und Ergebnisinterpretation

Die Hauptlimitierung dieser Arbeit stellt die retrospektive Datenerfassung dar. Die Dokumentation der untersuchten Variablen durch das Krebsregister, z. B. des AEG-Typs nach Siewert, hat erst im Laufe der Zeit zugenommen. Daher musste ein großer Teil des Grundkollektivs aufgrund fehlender Angaben ausgeschlossen werden (Abb. 2). Zudem konnte für die Variablen Grading sowie Lymphgefäß- und Veneninvasion bei einem nicht unerheblichen Anteil der Patienten keine Aussage getroffen werden. Daher kann der Effekt, den diese Variablen auf das Outcome besitzen, nicht abschließend geklärt werden. Bei der Arbeit wurden keine strukturierten Nachsorgeuntersuchungen angewendet. Dies geht mit der Gefahr einher, dass Patienten möglicherweise ein Rezidiv entwickelt haben, welches bisher nicht symptomatisch und daher noch nicht diagnostiziert worden ist. Daher könnten die tatsächlichen Rezidivraten höher liegen, als in dieser Arbeit ermittelt. Darüber hinaus resultiert dies in einer geringen Fallzahl in den einzelnen Gruppen. Insbesondere in der Gruppe der TAE mussten teils Analysen mit sehr niedrigen Patientenzahlen durchgeführt werden. Daher erfolgte eine gesonderte Untersuchung der Kohorte „Diagnosejahr 2016 bis 2020“, die über 50 % der Ösophagektomiepatienten erfasst. Eine Erklärung für die mögliche Tendenz innerhalb der Subgruppe in Richtung TAE bezüglich des Gesamtüberlebens könnte darin liegen, dass über die letzten Jahre hinweg deren Fallzahl absolut und relativ gesehen zugenommen hat. Die höhere Fallzahl und die damit verbundene Erfahrung der Operateure könnten zu einem besseren Outcome nach Ösophagektomie geführt haben. Die TEG dagegen gilt schon länger als etablierte Operationsmethode in der Therapie des AEG Typ II, weshalb im zeitlichen Verlauf nahezu keine Veränderungen bezüglich des Gesamtüberlebens detektiert werden konnten. Zwar ermöglicht die Untersuchung der Subgruppe die Betrachtung eines homogeneren Beobachtungszeitraums, allerdings gilt es zu berücksichtigen, dass das Follow-up dabei teilweise erheblich kürzer ist als im Gesamtkollektiv. Infolgedessen könnten potenzielle Todesereignisse bzw. Rezidive noch nicht aufgetreten sein. Somit bleibt die beschriebene mögliche Tendenz bezüglich des Gesamtüberlebens zugunsten der TAE kritisch zu betrachten.

### Schlussfolgerung

Zusammenfassend lässt sich festhalten, dass es in unserem Kollektiv keine signifikanten Unterschiede zwischen transhiatal erweiterter Gastrektomie und thorakoabdomineller Ösophagektomie in der Therapie des AEG Typ II gibt. Dabei müssen die oben diskutierten Limitierungen der Studie sowie deren mögliche Auswirkungen auf das Outcome der Patienten berücksichtigt werden.

Zudem gilt es, für beide Eingriffe die postoperative Mortalität bundesweit zu reduzieren. Möglicherweise wird eine durch die Mindestmengen versursachte Zentralisierung dabei eine positive Entwicklung triggern [16]. Dazu beitragen wird auch der häufigere Einsatz minimal-invasiver Operationstechniken [17].

Für eine definitive Festlegung auf eine der Operationsmethoden als chirurgisches Standardverfahren in der Therapie der AEG Typ II, werden die Ergebnisse größerer prospektiver Studien benötigt. Seit August 2019 läuft mit dem CARDIA-Trial des Uniklinikums Köln die erste internationale prospektive Studie, die sich mit dieser Thematik befasst [18].

## Fazit für die Praxis

- In den letzten 10 Jahren gab es eine absolute und relative Zunahme der TAE.
- Es bestehen keine Unterschiede zwischen TEG und TAE hinsichtlich der Operationskomplikationen.
- Es ergaben sich keine Unterschiede zwischen TEG und TAE hinsichtlich der postoperativen Mortalität.
- Es lagen keine Unterschiede zwischen TEG und TAE bezüglich des Gesamtüberlebensvor.
- Es besteht ein signifikanter Unterschied bezüglich der 5‑Jahres-Rezidivraten zugunsten der TEG.
- Es wurden keine Unterschiede zwischen TEG und TAE bezüglich des rezidivfreien Überlebens festgestellt.
